# CD117 Is a Specific Marker of Intraductal Papillary Mucinous Neoplasms (IPMN) of the Pancreas, Oncocytic Subtype

**DOI:** 10.3390/ijms21165794

**Published:** 2020-08-12

**Authors:** Paola Mattiolo, Seung-Mo Hong, Gaetano Paolino, Borislav C. Rusev, Giovanni Marchegiani, Roberto Salvia, Stefano Andrianello, Paola Capelli, Paola Piccoli, Claudia Parolini, Aldo Scarpa, Rita T. Lawlor, Claudio Luchini

**Affiliations:** 1 Department of Diagnostics and Public Health, Section of Pathology, University of Verona, 37134 Verona, Italy; paolamattiolo@gmail.com (P.M.); gaetano.paolino01@gmail.com (G.P.); borislavchavdarov.rusev@univr.it (B.C.R.); paola.capelli@aovr.veneto.it (P.C.); paola.piccoli@aovr.veneto.it (P.P.); claudia.parolini@aovr.veneto.it (C.P.); aldo.scarpa@univr.it (A.S.); 2 Department of Pathology, ASAN Medical Center, Seoul 05505, Korea; smhong28@gmail.com; 3 Department of Surgery, the Pancreas Institute, University and Hospital Trust of Verona, 37134 Verona, Italy; giovanni.marchegiani@univr.it (G.M.); roberto.salvia@univr.it (R.S.); stefano.andrianello@gmail.com (S.A.); 4 ARC-Net Research Center, University and Hospital Trust of Verona, 37134 Verona, Italy; ritateresa.lawlor@univr.it

**Keywords:** pancreas, intraductal, IOPN, IPMN, oncocytic, c-kit

## Abstract

The intraductal oncocytic papillary neoplasm (IOPN) of the pancreas has been recognized by WHO classification as a unique intraductal papillary mucinous neoplasm (IPMN) category. IOPN is composed of oxyphil cells, usually expressing MUC5AC, MUC6, and Hep Par-1, and harboring *PRKACA*/*B* fusion genes as their genetic hallmark. Although IOPNs are associated with an infiltrative adenocarcinoma in up to 30% of cases, the survival rate after surgical resection approaches 100%. This highlights the importance of the correct IOPN diagnosis, above all in cases with an associated invasive component. In this study, the immunohistochemical expression of CD117 was investigated in 111 IPMNs, including 17 oncocytic, 45 gastric, 20 pancreatico-biliary, and 29 intestinal IPMNs. We also tested the expression of MUC5AC, MUC6, and Hep Par-1 in the IOPN cohort. CD117 positivity was significantly more frequent in IOPNs compared to the other IPMN subtypes (*p* < 0.0001). Furthermore, within IOPN, a lower or absent CD117, MUC5AC, MUC6, and Hep Par-1 expression tended to be associated with the presence of an infiltrative component. Our findings shed light into the biology of these complex lesions, which are confirmed to be a distinctive IPMN subtype; notably, CD117 emerged as a potential, additional tool in the differential diagnosis of IPMNs.

## 1. Introduction

The oncocytic subtype of intraductal papillary mucinous neoplasm (oncocytic-IPMN), also named intraductal oncocytic papillary neoplasm (IOPN), of the pancreas has been recognized by the current WHO classification as a unique category of pancreatic IPMN [1]. It is a cystic epithelial neoplasm composed of exophytic nodular projections lined by oncocytic glandular epithelium, growing inside a dilated pancreatic ductal tree [1,2,3,4,5,6].

IOPNs represent about 5% of all intraductal neoplasms of the pancreas and are characterized by peculiar epidemiology and pathological features. They are more frequently discovered in males in their seventh decade of life and usually involve the main pancreatic duct [7,8,9]. Histologically, the cystic lesion is composed of oncocytic cells, which show a granular cytoplasm rich in mitochondria. IOPNs typically show high-grade dysplasia, and an associated carcinoma is found in up to 30% of cases [1,3,4]. At immunohistochemistry, IOPN express mucins such as mucin 1 (MUC1), mucin 5AC (MU5AC), and mucin 6 (MUC6), and other markers including Hep Par-1 [1,10,11,12,13,14]. The molecular landscape of IOPN is distinctive among pancreatic tumors. Recent reports identified *PRKACA*/*B* fusion genes as the genetic hallmark of this lesion [15,16], which also harbor recurrent mutations in the *EPHA8* and *ERBB4* genes, and lack the most common genetic alterations that characterize pancreatic ductal adenocarcinoma and the other types of IPMN, including *KRAS* and *GNAS* somatic mutations [1,12,13,14].

Although IOPNs are associated with invasive carcinoma in up to 30% of cases, and local recurrence rate is up to 40%, survival outcome is extremely favorable even after a second resection, with a five-year disease survival rate approaching 100% [1,17]. This observation highlights the importance of a precise differential diagnosis with the other intraductal neoplasms, above all in the case of an associated invasive component. This issue is fundamental for the appropriate clinical management, including therapeutic strategies and follow-up.

Currently, IOPN diagnosis is performed with histological evaluation, but it should be further confirmed by immunohistochemistry for a definitive classification [1,2]. This is based on the presence of positive staining patterns for markers such as MUC5AC, which is usually positive in all IPMN subtypes and is helpful in rule out an intraductal tubulopapillary neoplasm (typically MUC5AC negative); MUC6, for the differential diagnosis with the other IPMN subtypes (usually MUC6 negative in particular gastric and intestinal subtypes); and Hep Par-1, a cytoplasmic marker which is also positive in hepatocellular carcinoma and other cancer types composed of cells rich in mitochondria [1,2]. Table 1 summarizes the different staining patterns of mucins in the whole spectrum of intraductal lesions of the pancreas.

The aim of this study was to investigate the possible role of CD117 as a new potential biomarker supporting the diagnosis of IOPN, and to generate insights into the biology of this tumor entity. We focused our study on this marker since CD117 has been described as a potential useful immunohistochemical marker for oncocytic neoplasms of other districts, such as kidneys and thyroids [18,19,20,21,22,23,24,25,26]. CD117 is transmembrane protein functioning as a receptor tyrosine kinase [18,19]. It is considered a stem cell marker and is currently of interest in the fields of pancreas embryology, regenerative medicine, and tumor therapeutics [18,19,20,21,22]. Although recent evidences have pointed out that it can be expressed in some cases of pancreatic ductal adenocarcinoma [21,22,23], little is known about its expression in IPMN and IOPN.

## 2. Results

**Figure 1:**
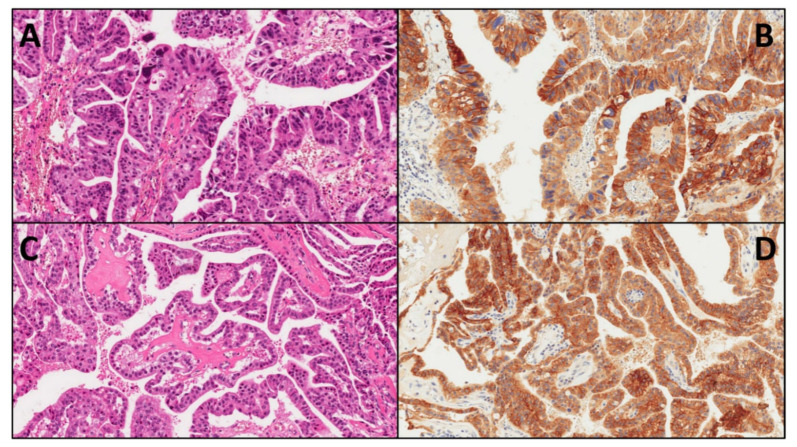
Representative cases of intraductal oncocytic papillary neoplasms (IOPN) of the pancreas with diffuse and strong CD117 positivity. (**A**,**B**). IOPN, area with high-grade dysplasia: hematoxylin–eosin (**A**), original magnification: 20×) and CD117 expression ((**B**), original magnification: 20×); histology shows nuclear atypia and granular, oxyphil cytoplasm (A); immunohistochemistry (B) shows the predominantly membranous staining pattern. (**C**,**D**). IOPN, area with low-grade dysplasia: hematoxylin-eosin (**A**), original magnification: 20×) and CD117 expression ((**B**), original magnification: 20×); the staining pattern was not influenced by the degree of dysplasia.

**Figure 2:**
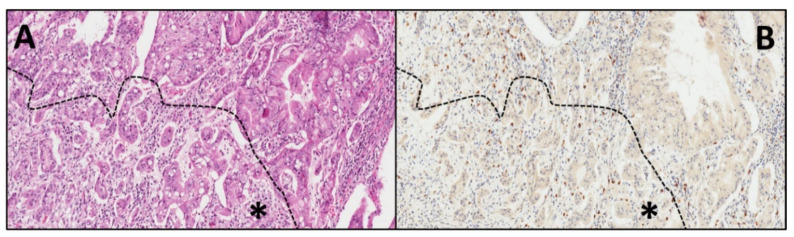
The CD117 negative IOPN had a non- infiltrating portion (upper part of the figures), and an invasive component (lower part, marked with an asterisk). Both intraductal and the invasive components were negative for CD117 (**A**): hematoxylin–eosin, (**B**): CD117 expression; original magnification: 20×). Scattered positive mast cells served as internal positive control (**B**).

Overall, 17 cases of IOPN were retrieved from the ARC-Net biobank at Verona University Hospital. All cases had high-grade dysplasia, and two cases showed an associated invasive carcinoma. Immunohistochemical analysis (IHC) on whole-section slides of IOPN showed positive results in 16/17 cases (94.1%). The staining pattern was predominantly membranous, with nuclei lacking positivity (Figure 1). The negative IOPN had an associated invasive component (Figure 2). Scattered mast cells, known to be CD117-reactive, were present in all cases and used as an internal positive control. Table 2 summarizes, for each IOPN, the immunostaining combined scores, which take into account both the percentage of positive cells (quantitative score) and the intensity of staining (qualitative score). The mean value of the combined score of all IOPNs was 5.47. Three cases showed the maximum combined score (12). The combined scores of the two cases with an associated invasive component were 0 and 1, lower values compared with those of non-invasive IOPNs, with a clearly different trend, although not statistically significant (mean combined scores of invasive IOPN vs. non-invasive IOPN: 0.5 in 2 cases vs. 5.93 in 15 cases, *p* = 0.09, *t*-Student test).

**Figure 3:**
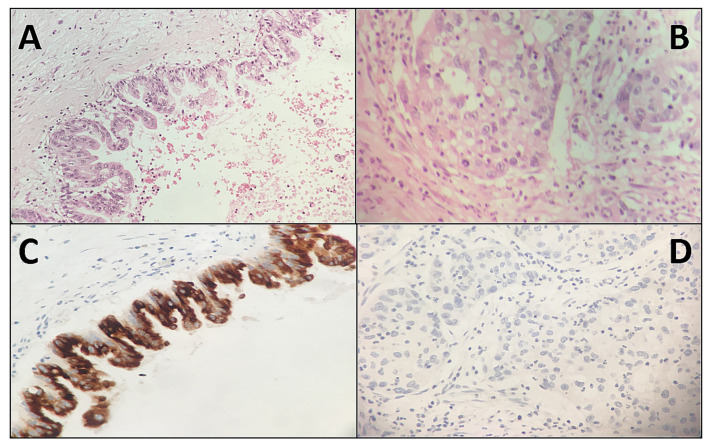
The expression of MUC5AC in the CD117 negative IOPN. It had a non-infiltrating portion (upper part of the figures), and an invasive component (lower part, marked with an asterisk). Both intraductal and the invasive components were negative for CD117 ((**A**) and (**B**): hematoxylin–eosin staining, where (**A**) represents the non-invasive component and (**B**) the infiltrative part; (**C**) and (**D**): MUC5AC staining, where (**C**) represents the non-invasive component and (**D**) the infiltrative part; original magnification: 10× (**A**) and (**B)**, 20× (**C**) and (**D**).

Table 3 summarizes the immunohistochemical results also for MUC5AC, MUC6, and Hep Par-1 in the 17 IOPNs. In particular, all these markers displayed a positivity in IOPNs, with a mean value of combined scores of 6.8 for MUC5AC, 6.5 for MUC6, and 6.8 for Hep Par-1. For intraductal lesions, there was not a statistical correlation between the staining patterns and their localization, of these markers and of CD117. Regarding IOPNs with an associated invasive component (case #2 and case #16), in the case with CD117 score 1, all these markers were positive in both the intraductal and invasive parts. However, in the case with CD117 score 0 (in both components), MUC5AC was positive only in the intraductal component, with loss of expression in the invasive part (Figure 3). At the same time, MUC6 and Hep Par-1 were positive in both components, but their expression was lower in the invasive part (Table 3).

The vast majority of non-oncocytic IPMNs were negative for CD117 (Table 4). In particular, 38/45 (84.4%) gastric IPMNs (36/43 with low grade dysplasia and 2/2 with high grade dysplasia), 15/20 (75%) pancreatico-biliary IPMNs (2/4 with low grade dysplasia and 13/16 with high grade dysplasia), and 26/29 (89.7%) intestinal IPMNs (14/16 with low grade dysplasia and 12/13 with high grade dysplasia) showed score 0. The mean combined score for all IPMNs was 0.29; the mean scores for each subtype were 0.27 for gastric IPMN, 0.5 for pancreatico-biliary IPMN, and 0.17 for intestinal IPMN.

In summary, CD117 resulted in being significantly more expressed in IOPNs compared with the other subtypes, considered together (16/17 vs. 15/94, *p* < 0.0001, Fisher’s exact test) and also separately (IOPN vs. gastric IPMN: 16/17 vs. 7/45, *p* < 0.0001; IOPN vs. pancreatico-biliary IPMN: 16/17 vs. 5/20, *p* < 0.0001; IOPN vs. intestinal IPMN: 16/17 vs. 3/29, *p* < 0.0001; Fisher’s exact test).

The differences between the mean values of the combined scores between IOPNs and the other IPMN subtypes were statistically significant, as follows: (i) IOPNs vs. all IPMNs: 5.47 vs. 0.29, *p* < 0.0001 (*t*-Student test); (ii) IOPNs vs. gastric IPMNs, 5.47 vs. 0.27, *p* < 0.0001 (*t*-Student test); (iii) IOPNs vs. pancreatico-biliary IPMNs, 5.47 vs. 0.5, *p* < 0.0001 (*t*-Student test); (iv) IOPNs vs. intestinal IPMNs, 5.47 vs. 0.17, *p* < 0.0001 (*t*-Student test).

## 3. Discussion

In this study, we tested the immunohistochemical expression of CD117 in 17 pancreatic IOPNs and in 94 cases of different pancreatic IPMN subtypes, including 45 gastric, 20 pancreatico-biliary, and 29 intestinal type IPMNs. The most important finding was the statistically significant differences between the number of CD117 positive cases of IOPNs vs. that of other types of IPMN (*p* < 0.0001), and between the mean combined IHC scores of IOPNs vs. that of other types of IPMN (*p* < 0.0001).

Our data clearly indicated that CD117 was expressed more frequently and with more diffuse/stronger staining patterns in IOPNs compared to the other IPMN subtypes [1,2,3]. This marker had been already described to be expressed in neoplasms composed of cells rich in mitochondria [1,24,25,26], and our findings confirmed this kind of association. Notably, CD117 did represent a potential additional tool to better distinguish IOPNs from the other IPMN subtypes. Currently, this distinction is carried out by morphology and using few reliable IHC markers, including MUC6 and Hep Par-1. However, the morphology of pancreatic intraductal neoplasms may be very heterogeneous, and the few IHC markers that can help in this distinction do not reach 100% of sensitivity and specificity. Based on our results, in selected cases, CD117 might be used as an additional immunohistochemical marker in a standard panel of antibodies for the differential diagnosis among the different types of pancreatic intraductal lesions. Along this line, the expression of MUC5AC, MUC6, and Hep Par-1 in our IOPN cohort was studied. Interestingly, although there was not a statistical correlation between the staining patterns of CD117 and of the other investigated markers for intraductal lesions, in the IOPN with a CD117 score 0, MUC5AC was positive only in the intraductal component, with a loss of expression in the invasive part. MUC6 and Hep Par-1 were positive in both components, but their expression was lower in the invasive part. These findings highlighted the biological differences between infiltrative and non-invasive components. This might reflect the lower differentiation of the invasive cells, which can lose the expression of some molecules during the carcinogenetic process. However, further studies are needed to better understand this complex oncogenetic process.

These results regarding CD117 expression appear of importance in further highlighting the biological differences between IOPNs and the other IPMN subtypes. IOPNs are confirmed to be a distinctive subtype among intraductal pancreatic neoplasms, also on the basis of CD117 expression. IOPNs represent a very peculiar category among pancreatic intraductal lesions, being associated with an excellent prognosis, with five-year disease survival rate approaching 100%. Oncocytic tumors are rare and show a unique biological behavior [24]. They can develop in the thyroid, parathyroid and salivary glands, kidneys, and adrenal cortex, and are almost invariably characterized by an indolent neoplastic behavior regardless of the site [25,26]. IOPNs share similar characteristics as, even if malignancy evolution is remarkable, adjuvant therapies and surgery for disease recurrence ensure an excellent prognosis, different from other IPMN subtypes [27]. The differential diagnosis of IOPNs vs. other IPMN subtypes is crucial in planning differentiated follow-up strategies. Patients should be informed about the high risk of recurrence (even several years after surgery), the favorable result of reoperation, and the need for a lifelong follow-up.

Interestingly, the combined scores of CD117 expression in the cases with an associated invasive component were lower when compared with those of non-invasive IOPNs, with a clearly different trend. This finding, although derived from a small sample size (only two infiltrative cases), merits further analysis, since a low/absent CD117 expression should be associated with a higher risk of malignant transformation in IOPNs. Although the pathological assessment of an eventual stromal invasion is based on histology, this might represent an interesting biological insight for a better comprehension of IOPN behavior.

In conclusion, this study demonstrated that, among the different IPMN subtypes, CD117 expression was strongly correlated with the oncocytic variant. This result indicated that CD117 might be used as a potential useful marker in differentiating IOPNs from the other IPMN subtypes and shed new light into the biology of these rare intraductal lesions. The specific expression of this marker in IOPNs further highlighted their peculiarity among all IPMN subtypes. Furthermore, the lower/absent CD117 expression observed in cases with an associated invasive component represents an interesting finding which should be further investigated in larger cohorts.

## 4. Materials and Methods

This study was approved by the local Ethical Committee (project: MN-2019, number of approval: 2610/CESC), and was conducted in accordance with the Good Practice guidelines, the Declaration of Helsinki and current laws, ethics, and regulations.

All cases with a diagnosis of IOPN or of oncocytic IPMN were retrieved from the ARC-Net biobank at Verona University Hospital. Only cases for which tumor slides and blocks were available were selected for this study. Furthermore, to compare the results of the immunohistochemical analysis obtained on whole sections of IOPNs with the other IPMN subtypes, we used tissue microarrays (TMA) of IPMNs with at least 3 cores per case. In total, the TMA included 94 cases: 45 gastric IPMNs (43 with low-grade dysplasia and 2 with high-grade dysplasia), 20 pancreatico-biliary IPMNs (4 with low-grade dysplasia and 16 with high-grade dysplasia), and 29 intestinal IPMNs (16 with low-grade dysplasia and 13 with high-grade dysplasia).

Immunohistochemical analysis (IHC) was performed as already described [28,29,30]. Briefly, four μm formalin-fixed and paraffin-embedded sections were immunostained with CD117 antibody (rabbit polyclonal, 1:100 dilution, Dako/USA). For IOPNs, one representative whole-section was used for each case. For TMA, at least three cores per case were evaluated. Heat-induced antigen retrieval was performed using a heated plate and 0.01 mol/L of citrate buffer, pH 8.9, for 15 min. Light nuclear counterstaining was performed with hematoxylin. All samples were processed using a sensitive peroxidase-based Bond polymer Refine detection system in an automated Bond instrument (Vision-Biosystem, Leica, Milan, Italy). Sections incubated without the primary antibody served as negative controls. We further studied the series of IOPN in the same way as described above, and with the following antibodies: Hep Par-1 (clone: OCH1E5, 1:50 dilution, Dako/USA), MUC5AC (clone: CLH2, 1:100 dilution, Novocastra/Germany), MUC6 (clone: CLH5, 1:100 dilution, Abnova/Taiwan).

IHC were evaluated separately and blindly by two residents in pathology (P.M., G.P.) and then reviewed by two pancreatic pathologists (A.S., C.L.). Inconsistencies were resolved by consensus at a multi-headed microscope. IHC was considered positive when the cell membrane of neoplastic cells stained for CD117. The overall evaluation was made using a combined quantitative and qualitative score.

First, the percentage of positive cells was calculated by assigning a quantitative score, as follows: no positive cells = 0, 1–25% = 1, 26–50% = 2, 51–75% = 3, 76–100% = 4. Then, a qualitative evaluation was performed, assigning a score based on the staining intensity: score 0 = no staining, score 1 = weak staining, score 2 = moderate staining, score 3 = strong staining. Finally, the combined score was calculated by multiplying the quantitative and the qualitative scores, with the final score ranging from 0 to 12. A positive combined score was considered when each score ≥1.

## Figures and Tables

**Table 1:** Immunohistochemical Mucins-Markers for IPMN/IOPN/ITPN Classification.

Types of Lesion	Subtype	MUC1	MUC2	MUC5AC	MUC6
IPMN	PB	positive	negative	positive	positive
	INT	negative	positive	positive	negative
	GAS	negative	negative	positive	negative
IOPN		positive	negative	positive	positive
ITPN		positive	negative	negative	positive

Abbreviations: IPMN: intraductal papillary mucinous neoplasm; IOPN: intraductal oncocytic papillary neoplasm; ITPN: intraductal tubulo-papillary neoplasm; G: gastric; PB: pancreatico-biliary; INT: intestinal.

**Table 2:** Summary of the Immunohistochemical Results for CD117 of the 17 Intraductal Oncocytic Papillary Neoplasms (IOPN) of the Pancreas. The Final Combined Score was Obtained by Multiplying the Quantitative and the Qualitative Scores. The Asterisk Indicates the Two IOPN With an Associated Invasive Component.

Case ID	Quantitative Score	Qualitative Score	Combined Score
1	1	1	1
2*	1	1	1
3	2	1	2
4	1	2	2
5	3	3	9
6	3	3	9
7	3	1	3
8	2	1	2
9	4	3	12
10	3	2	6
11	4	3	12
12	4	3	12
13	4	2	8
14	4	2	8
15	2	1	2
16 *	0	0	0
17	2	2	4

**Table 3:** Summary of the Immunohistochemical Results for CD117, MUC5AC, MUC6 and Hep Par-1 of the 17 Intraductal Oncocytic Papillary Neoplasms (IOPN) of the Pancreas. The Asterisk Indicates the two IOPN with an Associated Invasive Component.

Case ID	CD117	MUC5AC	MUC6	Hep Par-1
1	1	8	8	4
2 *	1 (1)	9 (9)	8 (8)	8 (8)
3	2	6	8	9
4	2	12	9	6
5	9	8	4	6
6	9	2	2	6
7	3	8	4	6
8	2	12	8	8
9	12	4	8	6
10	6	2	2	6
11	12	6	8	4
12	12	2	6	6
13	8	8	8	12
14	8	8	6	9
15	2	8	12	4
16 *	0 (0)	8 (0)	8 (4)	8 (2)
17	4	4	2	8

**Table 4:** Summary of Immunohistochemical Combined Scores of the Cohort of 94 Intraductal Papillary Mucinous Neoplasms (IPMN) of the Pancreas. Each Combined Score Indicates the Number of Positive Cases.

IPMN Subtype	Grade of Dysplasia	Combined Scores									N. of Cases
		0	1	2	3	4	6	8	9	12	
**PB**	LG	2	2	0	0	0	0	0	0	0	4
	HG	13	2	0	0	0	1	0	0	0	16
**INT**	LG	14	0	2	0	0	0	0	0	0	16
	HG	12	1	0	0	0	0	0	0	0	13
**GAS**	LG	36	3	3	1	0	0	0	0	0	43
	HG	2	0	0	0	0	0	0	0	0	2

Abbreviations: PB: pancreatico-biliary; INT: intestinal; GAS: gastric; LG: low-grade dysplasia; HG: high-grade dysplasia.

## Abbreviations

IPMN	Intraductal papillary mucinous neoplasm
IOPN	Intraductal oncocytic papillary neoplasm
WHO	world health organization
